# Supplementary material for: Multi‐dataset identification of innovative feature genes and molecular mechanisms in keratoconus
Source: J Cell Mol Med. 2024 Sep 19;28(18):e70079. doi: 10.1111/jcmm.70079 (PMC11412914; doi:10.1111/jcmm.70079)
Supplement: Supplementary file 2 — Material S2. [file JCMM-28-e70079-s002.zip › 7.GSEA analysis/4.MYRF/KEGG_TIGHT_JUNCTION.html]

Details for gene set KEGG\_TIGHT\_JUNCTION[GSEA]

|  || Dataset | MYRF.group.cls#h\_versus\_l |
| Phenotype | group.cls#h\_versus\_l |
| Upregulated in class | l |
| GeneSet | KEGG\_TIGHT\_JUNCTION |
| Enrichment Score (ES) | -0.40396807 |
| Normalized Enrichment Score (NES) | -1.4615577 |
| Nominal p-value | 0.028169014 |
| FDR q-value | 0.21995357 |
| FWER p-Value | 0.811 |
Table: GSEA Results Summary

  

Fig 1: Enrichment plot: KEGG\_TIGHT\_JUNCTION      
 Profile of the Running ES Score & Positions of GeneSet Members on the Rank Ordered List

  

| SYMBOL | TITLE | RANK IN GENE LIST | RANK METRIC SCORE | RUNNING ES | CORE ENRICHMENT || 1 | CLDN22 | na | 211 | 0.707 | 0.0308 | No |
| 2 | CLDN5 | na | 3852 | 0.259 | -0.0609 | No |
| 3 | MYH11 | na | 4047 | 0.252 | -0.0533 | No |
| 4 | RAB3B | na | 4294 | 0.245 | -0.0476 | No |
| 5 | MYH15 | na | 5198 | 0.216 | -0.0625 | No |
| 6 | EPB41 | na | 5239 | 0.215 | -0.0524 | No |
| 7 | CLDN18 | na | 6240 | 0.185 | -0.0716 | No |
| 8 | ASH1L | na | 7280 | 0.157 | -0.0935 | No |
| 9 | EPB41L3 | na | 7356 | 0.154 | -0.0876 | No |
| 10 | SYMPK | na | 7522 | 0.150 | -0.0846 | No |
| 11 | AMOTL1 | na | 8134 | 0.135 | -0.0952 | No |
| 12 | CLDN20 | na | 8679 | 0.123 | -0.1045 | No |
| 13 | EPB41L1 | na | 9079 | 0.114 | -0.1100 | No |
| 14 | EXOC3 | na | 9194 | 0.112 | -0.1075 | No |
| 15 | KRAS | na | 9371 | 0.108 | -0.1069 | No |
| 16 | CLDN7 | na | 9372 | 0.108 | -0.1013 | No |
| 17 | CLDN3 | na | 9491 | 0.106 | -0.0992 | No |
| 18 | GNAI3 | na | 9952 | 0.097 | -0.1074 | No |
| 19 | AKT2 | na | 10210 | 0.092 | -0.1100 | No |
| 20 | PPP2R2B | na | 10242 | 0.092 | -0.1062 | No |
| 21 | CSNK2A1 | na | 10382 | 0.089 | -0.1055 | No |
| 22 | MYH14 | na | 10451 | 0.087 | -0.1029 | No |
| 23 | MYL5 | na | 10477 | 0.087 | -0.0991 | No |
| 24 | EXOC4 | na | 10719 | 0.083 | -0.1017 | No |
| 25 | OCLN | na | 10725 | 0.083 | -0.0976 | No |
| 26 | MYH13 | na | 10964 | 0.079 | -0.1003 | No |
| 27 | SPTAN1 | na | 11089 | 0.077 | -0.0999 | No |
| 28 | MYH6 | na | 11121 | 0.076 | -0.0968 | No |
| 29 | PTEN | na | 11228 | 0.075 | -0.0960 | No |
| 30 | LLGL2 | na | 11393 | 0.072 | -0.0970 | No |
| 31 | TJP2 | na | 11404 | 0.072 | -0.0935 | No |
| 32 | PPP2R2D | na | 12346 | 0.059 | -0.1177 | No |
| 33 | CLDN19 | na | 12427 | 0.058 | -0.1170 | No |
| 34 | ACTB | na | 12485 | 0.057 | -0.1157 | No |
| 35 | CLDN16 | na | 12551 | 0.056 | -0.1146 | No |
| 36 | CLDN23 | na | 12578 | 0.056 | -0.1125 | No |
| 37 | MYH7 | na | 12644 | 0.055 | -0.1115 | No |
| 38 | TJP1 | na | 12904 | 0.051 | -0.1163 | No |
| 39 | CLDN8 | na | 12946 | 0.051 | -0.1148 | No |
| 40 | CGN | na | 13240 | 0.047 | -0.1208 | No |
| 41 | YBX3 | na | 13452 | 0.044 | -0.1246 | No |
| 42 | CTTN | na | 13470 | 0.044 | -0.1228 | No |
| 43 | PRKCQ | na | 13573 | 0.043 | -0.1235 | No |
| 44 | MAGI3 | na | 13711 | 0.041 | -0.1253 | No |
| 45 | PARD6G | na | 13828 | 0.040 | -0.1266 | No |
| 46 | PRKCZ | na | 13995 | 0.038 | -0.1294 | No |
| 47 | PRKCA | na | 14202 | 0.036 | -0.1335 | No |
| 48 | MYH8 | na | 14456 | 0.033 | -0.1391 | No |
| 49 | CTNNA2 | na | 14571 | 0.032 | -0.1407 | No |
| 50 | PPP2R2C | na | 14588 | 0.032 | -0.1395 | No |
| 51 | ACTN4 | na | 14881 | 0.029 | -0.1465 | No |
| 52 | PPP2R1B | na | 15086 | 0.027 | -0.1509 | No |
| 53 | GNAI2 | na | 15256 | 0.025 | -0.1545 | No |
| 54 | MYH2 | na | 15413 | 0.024 | -0.1578 | No |
| 55 | CTNNB1 | na | 15600 | 0.022 | -0.1620 | No |
| 56 | MYL12B | na | 15972 | 0.018 | -0.1718 | No |
| 57 | PRKCG | na | 15973 | 0.018 | -0.1709 | No |
| 58 | PRKCI | na | 16307 | 0.015 | -0.1797 | No |
| 59 | PARD3 | na | 16431 | 0.013 | -0.1826 | No |
| 60 | LLGL1 | na | 16448 | 0.013 | -0.1823 | No |
| 61 | CTNNA1 | na | 16494 | 0.013 | -0.1830 | No |
| 62 | MYH10 | na | 17326 | 0.006 | -0.2067 | No |
| 63 | MYL12A | na | 18052 | -0.001 | -0.2276 | No |
| 64 | AKT1 | na | 18101 | -0.001 | -0.2289 | No |
| 65 | PRKCH | na | 18156 | -0.002 | -0.2304 | No |
| 66 | RAB13 | na | 18491 | -0.005 | -0.2397 | No |
| 67 | CLDN9 | na | 18587 | -0.006 | -0.2422 | No |
| 68 | F11R | na | 18606 | -0.006 | -0.2424 | No |
| 69 | RHOA | na | 18827 | -0.008 | -0.2483 | No |
| 70 | CSNK2B | na | 19992 | -0.022 | -0.2808 | No |
| 71 | MYH9 | na | 20365 | -0.026 | -0.2902 | No |
| 72 | CSNK2A2 | na | 20546 | -0.028 | -0.2939 | No |
| 73 | CRB3 | na | 20554 | -0.028 | -0.2926 | No |
| 74 | PRKCD | na | 20569 | -0.028 | -0.2916 | No |
| 75 | TJP3 | na | 20822 | -0.031 | -0.2972 | No |
| 76 | CASK | na | 21312 | -0.037 | -0.3094 | No |
| 77 | HRAS | na | 21856 | -0.044 | -0.3228 | No |
| 78 | VAPA | na | 21953 | -0.045 | -0.3232 | No |
| 79 | ACTG1 | na | 22317 | -0.050 | -0.3311 | No |
| 80 | PPP2R1A | na | 22646 | -0.054 | -0.3377 | No |
| 81 | PARD6A | na | 22656 | -0.054 | -0.3352 | No |
| 82 | YES1 | na | 23276 | -0.064 | -0.3497 | No |
| 83 | CDC42 | na | 23297 | -0.064 | -0.3470 | No |
| 84 | CLDN1 | na | 23552 | -0.068 | -0.3507 | No |
| 85 | CDK4 | na | 24674 | -0.088 | -0.3786 | No |
| 86 | CTNNA3 | na | 25302 | -0.099 | -0.3915 | No |
| 87 | CLDN4 | na | 25502 | -0.103 | -0.3919 | No |
| 88 | MYH3 | na | 25603 | -0.105 | -0.3893 | No |
| 89 | PRKCB | na | 25770 | -0.108 | -0.3885 | No |
| 90 | PPP2CB | na | 25840 | -0.109 | -0.3848 | No |
| 91 | PPP2R2A | na | 26095 | -0.115 | -0.3861 | No |
| 92 | RRAS2 | na | 26466 | -0.122 | -0.3904 | No |
| 93 | PPP2CA | na | 26826 | -0.131 | -0.3940 | No |
| 94 | CLDN15 | na | 27173 | -0.139 | -0.3967 | Yes |
| 95 | CLDN11 | na | 27203 | -0.140 | -0.3902 | Yes |
| 96 | RRAS | na | 27376 | -0.144 | -0.3877 | Yes |
| 97 | MYH1 | na | 27858 | -0.157 | -0.3934 | Yes |
| 98 | ACTN3 | na | 27890 | -0.157 | -0.3861 | Yes |
| 99 | ACTN1 | na | 27985 | -0.160 | -0.3804 | Yes |
| 100 | GNAI1 | na | 28414 | -0.173 | -0.3838 | Yes |
| 101 | MYL9 | na | 28537 | -0.176 | -0.3781 | Yes |
| 102 | CLDN10 | na | 28723 | -0.182 | -0.3739 | Yes |
| 103 | TJAP1 | na | 29031 | -0.192 | -0.3728 | Yes |
| 104 | MYH4 | na | 29629 | -0.211 | -0.3790 | Yes |
| 105 | NRAS | na | 30019 | -0.224 | -0.3786 | Yes |
| 106 | ACTN2 | na | 30149 | -0.229 | -0.3704 | Yes |
| 107 | MYL2 | na | 30601 | -0.246 | -0.3706 | Yes |
| 108 | MPDZ | na | 30930 | -0.260 | -0.3665 | Yes |
| 109 | SRC | na | 31183 | -0.273 | -0.3595 | Yes |
| 110 | CLDN17 | na | 31502 | -0.291 | -0.3535 | Yes |
| 111 | MYL7 | na | 31601 | -0.295 | -0.3409 | Yes |
| 112 | IGSF5 | na | 31655 | -0.298 | -0.3269 | Yes |
| 113 | HCLS1 | na | 32256 | -0.334 | -0.3268 | Yes |
| 114 | CLDN6 | na | 32462 | -0.348 | -0.3146 | Yes |
| 115 | MYH7B | na | 32705 | -0.366 | -0.3025 | Yes |
| 116 | MYL10 | na | 33204 | -0.407 | -0.2957 | Yes |
| 117 | MAGI2 | na | 33274 | -0.416 | -0.2759 | Yes |
| 118 | PARD6B | na | 33359 | -0.426 | -0.2561 | Yes |
| 119 | MAGI1 | na | 33417 | -0.433 | -0.2352 | Yes |
| 120 | PRKCE | na | 33586 | -0.454 | -0.2164 | Yes |
| 121 | EPB41L2 | na | 33622 | -0.459 | -0.1934 | Yes |
| 122 | CLDN2 | na | 34148 | -0.557 | -0.1795 | Yes |
| 123 | JAM2 | na | 34223 | -0.578 | -0.1515 | Yes |
| 124 | CLDN14 | na | 34256 | -0.588 | -0.1218 | Yes |
| 125 | MRAS | na | 34392 | -0.631 | -0.0927 | Yes |
| 126 | AKT3 | na | 34696 | -0.932 | -0.0529 | Yes |
| 127 | JAM3 | na | 34714 | -1.030 | 0.0004 | Yes |
Table: GSEA details [plain text format]

  

Fig 2: KEGG\_TIGHT\_JUNCTION      
 Blue-Pink O' Gram in the Space of the Analyzed GeneSet

  

Fig 3: KEGG\_TIGHT\_JUNCTION: Random ES distribution      
 Gene set null distribution of ES for **KEGG\_TIGHT\_JUNCTION**
